# Genomic Prediction Using Canopy Coverage Image and Genotypic Information in Soybean via a Hybrid Model

**DOI:** 10.1177/1176934319840026

**Published:** 2019-03-29

**Authors:** Reka Howard, Diego Jarquin

**Affiliations:** 1 Department of Statistics, University of Nebraska–Lincoln, Lincoln, NE, USA; 2 Department of Agronomy and Horticulture, University of Nebraska–Lincoln, Lincoln, NE, USA

**Keywords:** Prediction, molecular marker, canopy coverage, hybrid matrix model, clustering, cross-validation, SoyNAM

## Abstract

Prediction techniques are important in plant breeding as they provide a tool for selection that is more efficient and economical than traditional phenotypic and pedigree based selection. The conventional genomic prediction models include molecular marker information to predict the phenotype. With the development of new phenomics techniques we have the opportunity to collect image data on the plants, and extend the traditional genomic prediction models where we incorporate diverse set of information collected on the plants. In our research, we developed a hybrid matrix model that incorporates molecular marker and canopy coverage information as a weighted linear combination to predict grain yield for the soybean nested association mapping (SoyNAM) panel. To obtain the testing and training sets, we clustered the individuals based on their marker and canopy information using 2 different clustering techniques, and we compared 5 different cross-validation schemes. The results showed that the predictive ability of the models was the highest when both the canopy and marker information was included, and it was the lowest when only the canopy information was included.

## Introduction

Plant breeding is a science that uses multiple scientific disciplines to develop improved cultivars. Some of the scientific fields involved in plant breeding are genetics, agronomy, soil science, botany, biochemistry, plant pathology, computer science, statistics, and engineering. The goal of plant breeding is to combine the advantages of these disciplines to increase food security and face the environmental challenges, thus producing high-yielding cultivars with agronomically desired traits for humans in a sustainable way.

One of the biggest tasks of plant breeders is to select individuals for crossing that will produce the desired phenotype. Breeders have to cross individuals and test the progeny in several cycles. They not only measure their success regarding the amount of increase in the performance of the cultivar that is achieved in a cycle (called genetic gain) but also in the amount of time they can release a cultivar to the farmers. One of the ways in which the development process can be shortened is by decreasing the amount of cycles used to develop the cultivar. To achieve this goal, several methods were developed that help in the selection procedure. The development of inexpensive genotyping strategies such as genotype by sequencing (GBS) and single-nucleotide polymorphism (SNP)^1,2^ made it feasible for plant breeders to take advantage of molecular marker information and develop techniques that improve the efficiency of selection in plant breeding. Molecular markers were first used for prediction purposes in the 1970s when it was assumed that only a few genes affect the phenotype, and it is financially feasible to develop progeny with the desired genes. This approach is called quantitative trait locus (QTL) mapping.^3,4^ However, the success of QTL mapping depends on the number of QTLs affecting the phenotype. Agronomically important traits (eg, yield) usually are influenced by many small-effect QTLs instead of by few large-effect QTLs. As a result, the adoption of the technology into breeding programs is problematic because it is very expensive to develop a population using this method.

Another method that takes advantage of the dense molecular marker information is called genomic prediction (GP) or genomic selection (GS). The distinction between the 2 terms relies on the notion that genomic information is used for predicting the phenotype, and the prediction results are used by the plant breeders to select individuals for advancing into the next generation and make the desired crosses. Although there are earlier articles describing the foundation of this method,^5–7^ the first article defining GS using dense markers was presented by Meuwissen et al.^8^ Since then, there has been a large amount of effort dedicated to developing methods for GP and evaluating them under various conditions.^9–13^ GP has the potential to increase the expected genetic gain by reducing the cycle length, and it uses fewer resources compared with traditional phenotypic selection; thus, it has the ability to improve complex traits that are influenced by many small-effect QTLs and their interactions, and traits with low heritability. Evidently, the improvement is more significant for simple traits that are controlled by fewer QTLs and traits with high heritability.^14^

Phenomics is an area of research that opens new avenues in the field of prediction for plant breeding. With this new technique, physical and biochemical traits of the plants can be measured in a cost-effective way, and the measurements can be monitored over time. However, it also introduces new challenges in plant breeding because an extensive amount of data can be collected, and the existing statistical methods have difficulty incorporating the large dimensionality of the collected data. Thus, new statistical techniques and algorithms have to be developed to efficiently use the given data to increase the predictive ability of complex traits.

In this article, we introduce a model that combines molecular marker and canopy coverage image information via a combined relationship matrix for the soybean nested association mapping (SoyNAM) population. Christensen et al^15^ and Legarra et al^16^ have developed similar models by combining marker and pedigree data. To our knowledge, this is the first time that phenotypic and genomic data are used to compute a hybrid matrix. For the model, we create a hybrid matrix that combines both the molecular marker and canopy coverage data. We predict the yield of the lines and compare the predictive ability of the model with that of the models where only the molecular marker information and only the canopy coverage information are included. The ultimate goal is to help improve the selection tools used by breeders. In this article, first, we introduce the SoyNAM population and discuss the structure, composition of the population, and molecular marker and canopy data. Then, we discuss how the hybrid matrix is developed from the base GP model. For the prediction, we considered 5 different ways to split the data into training and testing sets based on 2 clustering algorithms. We describe the clustering algorithms used to divide the data into testing and training sets, and how the data were divided. Finally, we present the results and some future avenues to improve the model.

## Material and Methods

### The SoyNAM dataset

For the evaluation of our model, we used data collected from the SoyNAM population (https://www.soybase.org/SoyNAM/), which is a nested association mapping population originally consisting of 5600 F5-derived recombinant inbred lines (RILs). The RILs were derived by crossing a common, high-yielding parent (IA3023) to 40 other parents. Out of the 40 parents, 17 were high-yielding, elite lines from 8 different states from the United States, 15 lines had diverse ancestry, and 8 were considered as exotic lines as their origins were South Korea, China, Russia, and Serbia. Originally, the development of the SoyNAM population started in 2011, but the phenotypic and canopy coverage data used in this study were observed in 2013.^17^ Details of the experimental design can be found in Xavier et al.^17^ The molecular marker information consisted of 5305 SNP markers but, after employing quality control (removing markers with minor allele frequency less than 0.05), about 4600 SNP markers were included into the model.^17^

Canopy coverage information, which is the area covered by the plant, can easily be measured on soybean plants. The canopy coverage is correlated with canopy light interception, which is a trait that is positively correlated with grain yield but difficult to measure.^18^

The canopy image data used for evaluating our model were ground-based red-green-blue (RGB) images. Then, the raw images were converted into a metric that describes the percentage of image pixels that are classified as canopy pixels. The images were collected at regular intervals from 2 to 8 weeks after planting; thus, only 6 measurements were obtained. To extrapolate the data, allowing estimations of daily measures, a logistic growth function via the logit link was used to model longitudinal data of canopy coverage for each genetic line. In this way, at the end for each RIL, there were (6 × 7) 42 canopy coverage measurements (14-56 days after planting) available. The data and models employed in this research are described in detail by Xavier et al.^17^

The phenotypic trait that we predicted using the molecular marker and canopy coverage information was grain yield (in kg/ha). There are 8 other traits that were observed for the SoyNAM population, but we only considered the agronomically most important trait. For more information about the development of the SoyNAM population, the genotypic, phenotypic, and canopy coverage information, the reader can refer to Xavier et al.^17,19^

### The hybrid matrix model development

The goal of this study was to develop a model to predict grain yield that incorporates molecular marker and canopy coverage image information using the SoyNAM population. To build a hybrid matrix model, the following model was used as a base

$$y_{i}=\mu +u_{i}+\varepsilon_{i}$$

where $y_{i}$ is the phenotypic response of the *i*^th^ (i=1,2,…,n) genotype and it can be explained as the sum of a common mean (m), plus random deviations due to genetic effects $\left(u_{i}\right)$, plus an error term $\left(\varepsilon_{i}\right)$, where $u=\{u_{i}\}∼N(0,G\sigma_{u}^{2})$ with G acting as a covariance structure that describes similarities of different types (genetic via molecular markers, phenotypic via daily measures of canopy values, or both) between pairs of individuals, and $\sigma_{u}^{2}$ is the associated variance component. This model, also known as genomic best linear unbiased prediction (GBLUP) model, was introduced in the GS context following standard assumptions of the Ridge-Regression-BLUP model for the marker effects as identically and independently distributed draws from a normal distribution. Details of this model can be found in Habier et al^20^ and VanRaden.^21^

Because the marker and canopy data were available, these 2 sources of information were used for computing covariance structures as follows: $G_{X}=XX^{\prime }/p$ and $G_{C}=CC^{\prime }/q$ with $X_{n\times p}$ and $C_{n\times q}$ as the centered and standardized (by columns) matrices for *n* (5600) phenotypic records, *p* (5305) genomic markers, and *q* (42) canopy records derived from the image data. In this case, the standardization by columns is not needed; however, it allows interpreting the variance components as genomic and image variances in the same scale relative to the error variance. Initially, the marker values were coded based on the number of copies of the allele with the minor allele frequency (0, 1, and 2). In the case of the canopy data, these values ranged between 0 and 1 to indicate the proportion of covered area of each plot by the canopy vegetation. In both cases, these values were standardized (centered at 0 and with a unit variance). Thus, after standardization, these values of both sources of information had the same scale.

Here, $G_{X}$ and $G_{C}$ have the same dimensions (*n* × *n*) allowing the cell-to-cell additive operations.

The hybrid matrix could be built combining both covariance structures as a linear combination that depends on the weight (w) where 0≤w≤1

$$G_{F}=G_{X}\cdot \left(1-w\right)+G_{C}\cdot \left(w\right)$$

In this study, values between 0 and 1 in steps of 0.01 were used for w, such that w={0,0.1,0.2,0.3,0.4,0.5,0.6,0.7,0.8,0.9,1}, and these values were tested in a grid search for optimizing the predictive ability of the proposed models.

### Cross-validation methods

To evaluate the prediction models, 3 cross-validation (CV) schemes were employed. The first one was the leave-one-observation-out CV where, for predicting an unobserved genotype, we used all of the remaining RILs. The other ones were based on 2 clustering techniques. We clustered the RILs based on the Euclidean distance among the combined molecular marker and canopy coverage information. We compared 2 clustering algorithms: Clustering Large Applications (CLARA) and a Hierarchical Linkage (HL) method.

CLARA^22^ is based on *k*-means clustering technique^23^ which is an unsupervised machine learning technique that divides the data into *k* clusters (groups) based on the Euclidean distance between the individuals (in our case the molecular markers and the canopy coverage information of the RILs). CLARA extends the idea of the k-means clustering, and it divides on the data into k clusters using a sampling approach. CLARA is an iterative procedure where first a small sample of the data is selected, and then the clustering is performed. This procedure is repeated a pre-specified number of times. The clustering within the samples is done by first finding the central objects of the clusters, then assigning all of the other observations to the nearest central object based on the Euclidean distance. The clustering is finalized based on the minimum of the sum of the dissimilarities of the objects to the nearest central object.^24^

HL method is a clustering technique that is based on the dissimilarity of the objects, and it builds the clusters starting with the objects being individual clusters (which also called as a “bottom-up” approach). The dissimilarity of the objects is based on the Euclidean distance among the molecular markers and the canopy coverage information of the RILs. For dividing the objects into clusters, the Ward method was implemented which minimizes the total within-cluster variance.

Essentially, the foundations of methods for creating clusters are different. Whereas CLARA attempts to find clusters based on similarities within randomly selected subsets of the data, the HL method starts with single-element clusters and builds the clusters in a “bottom-up” manner.

For both the CLARA and the HL methods, the RILs were partitioned into 2, 3, 4, and 5 clusters. The predictions were performed using the leave-one-observation-out CV within each of the 2, 3, 4, or 5 clusters. Once the predictions for all clusters were computed, these values were integrated into a single vector across clusters. Then, the predictive ability for each of the 99 strategies was evaluated using the Pearson correlation coefficient between the entire vectors of the observed and the predicted values.

## Results and Discussion

In this study, we developed a hybrid matrix approach for incorporating molecular marker and canopy coverage information to predict grain yield for the SoyNAM population. For the hybrid matrix, we evaluated 11 different weights. One of the extremes was when absolutely no canopy coverage information was included in the training-testing sets and these were composed only of marker data. The other extreme was the opposite case, where no marker information was used but only canopy coverage information was available. The intermediate cases had both canopy and marker information at different intensities. We clustered the RILs using the CLARA and HL methods, and the numbers of clusters we considered were 2, 3, 4, and 5. Within each cluster, for dividing the data into training and testing sets, we used the leave-one-observation-out CV scheme, where 1 RIL was predicted using all of the remaining RILs. The predictive ability was evaluated based on the correlation between the observed and the predicted grain yield.

We considered prediction strategies based on the combination of 11 weights *w* (ranging between 0 and 1 in increments of 0.1, such that *w* = 0, 0.1, 0.2, . . ., 1), 2 clustering techniques (CLARA and HL), and 4 different cluster sizes (2, 3, 4, 5) which resulted in 88 unique combinations (11 weights × 2 clustering techniques × 4 cluster sizes). Also, the case where no clusters were formed (i.e., the whole population is the unique cluster) was considered, generating another 11 combinations (the weights). Thus, there were a total of 99 (88 + 11) unique combinations considered in this study. The results of the previous combinations are shown in Table 1.

**Table 1.** Predictive ability values for the CLARA and HL methods using 2, 3, 4, and 5 clusters compared with the leave-one-observation-out CV for the different weights of the hybrid matrix model.

CLUSTER	1	2		3		4		5	
METHOD WEIGHT	NONE	CLARA	HL	CLARA	HL	CLARA	HL	CLARA	HL
0	0.531	0.506	0.506	0.496	0.49	0.477	0.472	0.466	0.463
0.1	0.599	0.596	0.593	0.588	0.575	0.576	0.566	0.568	0.554
0.2	0.599	0.592	0.595	0.598	0.594	0.584	0.581	0.578	0.571
0.3	0.598	0.589	0.603	0.599	0.597	0.587	0.589	0.575	0.575
0.4	0.598	0.592	0.598	0.588	0.599	0.589	0.58	0.576	0.57
0.5	0.599	0.597	0.597	0.596	0.583	0.587	0.576	0.573	0.571
0.6	0.598	0.592	0.602	0.597	0.599	0.587	0.587	0.575	0.578
0.7	0.598	0.597	0.589	0.598	0.585	0.586	0.583	0.577	0.578
0.8	0.599	0.595	0.597	0.6	0.596	0.587	0.587	0.58	0.572
0.9	0.599	0.597	0.601	0.597	0.598	0.587	0.585	0.572	0.574
1	0.276	0.308	0.302	0.308	0.304	0.315	0.308	0.314	0.312

CLARA, Clustering Large Applications; CV, cross-validation; HL, Hierarchical Linkage.

From Table 1, we can see that the highest predictive ability when no clusters were considered (i.e., only 1 cluster, the entire population) was 0.599. This value was obtained for different weights *w* (0.1, 0.2, 0.5, 0.8, and 0.9). Regarding the efficiency of the 2 clustering techniques to improve the predictive ability, we observed that for the same cluster size × weight combinations these results were very similar. However, the highest correlation among all cluster sizes was 0.603, and it was reached using the HL cluster method for creating 2 clusters with a *w* value of 0.3 (i.e., 30% of importance was given to marker data and the remaining 70% to canopy image data). However, this value was not significantly different from other high values that were found for other *w* values. For example, for this same clustering method (HL) and cluster size, the predictive abilities of 0.602 and 0.601 were obtained for the *w* values of 0.6 and 0.9, respectively. Thus, no specific patterns about the importance of marker-canopy data for improving the predictive ability were found. For the case when 3 clusters were considered, the highest predictive abilities were 0.6 and 0.599 for the strategies (CLARA with *w* = 0.8) and (HL with *w* = 0.4, 0.6), respectively. With 4 clusters, CLARA with *w* = 0.4 and HL with *w* = 0.3 produced the highest correlation (0.589). Finally, with 5 clusters, the highest values were 0.58 (CLARA with *w* = 8) and 0.578 (HL with *w* = 0.6, 0.7). Although no significant improvements in predictive ability were shown by constructing homogeneous clusters, a slight increase in the correlation was observed when solely canopy data were used (i.e., *w* = 1). These values ranged between 0.276 (for only 1 cluster) and 0.314 (5 clusters using CLARA). On the other hand, the predictive ability was reduced with clustering when only marker data were included (i.e., *w* = 0). These values ranged between 0.531 (no clusters) and 0.463 (5 clusters using HL).

In the figures, we only show the results using the CLARA method due to the similarity of the clustering techniques regarding predictive ability. The top row of the figures shows the correlation plots when the leave-one-observation-out CV is implemented for the whole dataset without clustering, and the bottom row of the figures shows the results for the CLARA method when 5 clusters were considered. The left panels are the correlation plots when only canopy coverage information is included (*w* = 1) into the prediction model but no molecular marker information, the center panels are the correlation plots when only molecular marker information (*w* = 0) is included into the model but no canopy coverage information, and the right panels are the correlation plots when both the marker and canopy information is included with an equal weight (*w* = 0.5). We only included the plots where *w* = 0.5, and not the other cases where 0 < *w* < 1 because the results were very similar (see Table 1).

**Figure 1.**
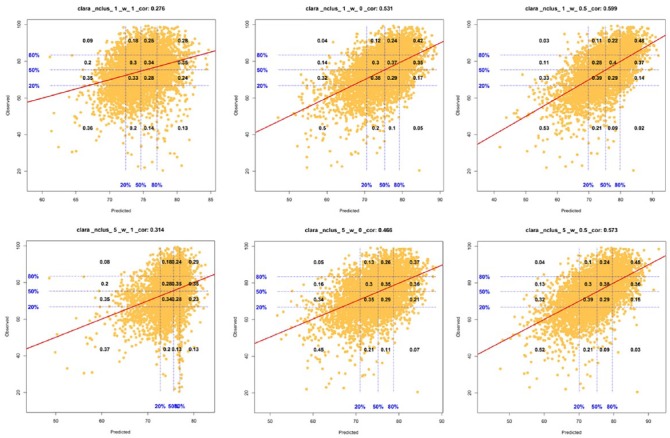
Scatter plots showing the prediction accuracy between the predicted (*x*-axis) and observed values (*y*-axis) using 2 cluster sizes (1—top row, 5—bottom row) and 3 different weight values (*w* = 0, 0.5, and 1). The CLARA method was used for designing the 5 clusters (bottom row). The correlation plots on the left represent the results for the models when only canopy coverage information is included, in the middle when only molecular marker information is included, and on the right when both canopy and marker information is included, and they are weighted the same (*w* = 0.5). The horizontal and vertical lines give the empirical percentiles (20%, 50%, and 80%) of the variables on the corresponding *x*-*y* axis and the numbers inside the grid provide the success rate of classification for each of the 4 groups defined by the percentiles displayed for the observed values, given the groups defined in the vertical line. For the case where 5 clusters were considered (bottom row), the predicted values for each cluster were integrated into a single vector and these were plotted against the observed values. In the same way, the correlation was computed using the entire vectors of the predicted and observed values. CLARA, Clustering Large Applications.

The plots in Figure 1 show the predictive ability as the correlation between the observed and predicted values of grain yield. The title of the plots includes the overall correlation coefficients (i.e., the prediction between the entire vector of the predicted and observed values across clusters), and the plots include the correct classification rates for 16 combinations between 4 different intervals based on empirical percentiles ([0%, 20%], [20%, 50%], [50%, 80%], and [80%, 100%]) for the predictions and 4 intervals ([0%, 20%], [20%, 50%], [50%, 80%], and [80%, 100%]) for the observed values. Within each plot, the top right value represents the proportion of the top 20% of the observed values that were also predicted as the top 20% in grain yield. On the other hand, if we were interested in screening or discarding the lowest 20% of the lines based on the predictions, the bottom left value shows the proportion of correctly discarded lines based on the observed values. In this case, the objective would be to increase the classification rate values in the diagonal grid of the plot and to reduce the classification error rate addressed in the off diagonal. In general, we observed that the classification rate in the diagonal of the grid improved when the marker and canopy image data were combined using the hybrid matrix and the classification error rate decreased (i.e., for cases in the off diagonal).

When we compare the overall correlation coefficients and the classification error rate (the numbers within the grid in the off diagonal), we observed that the predictive ability was the lowest when we only included canopy coverage information into the prediction model, and thus the classification error rate was larger than those in the other 2 cases. When only marker information was considered, the predictive ability was improved and the classification error rate was reduced.

Prediction techniques are valuable tools for plant breeders as they can evaluate a larger number of candidates and select a less number of lines with the same level of efficiency and less cost than with the traditional phenotypic selection; thus, it is important to improve the predictive ability of the available models. With the advancements of collecting image information on plants, we have the opportunity to develop prediction models where we integrate multiple sources of information. The hybrid matrix model can integrate the diverse information as a weighted linear combination of the different sources of information, and it increases the predictive ability compared with models that only incorporated a single source of variation into the model (e.g., only molecular marker or only canopy coverage information). In our future work, we plan to develop a model that also includes the interaction of the hybrid matrix with environmental covariates.

## References

[1] Elshire RJ Glaubitz JC Sun Q et al A robust, simple genotyping-by-sequencing (GBS) approach for high diversity species. PLoS ONE. 2011;6:e19379. doi:10.1371/journal.pone.0019379. PMC3087801 21573248

[2] Kumar S Travis W Banks TW Cloutier S. SNP discovery through next-generation sequencing and its applications. Intern J Plant Genom. 2012;2012:831460. doi:10.1155/2012/831460. PMC3512287 23227038

[3] Soller M Plotkin-Hazan J. The use of marker alleles for the introgression of linked quantitative alleles. Theor Appl Genet. 1977;51:133–137. 24317690 10.1007/BF00273825

[4] Soller M. The use of loci associated with quantitative effects in dairy cattle improvement. Anim Prod. 1978;27:133–139.

[5] Bernardo R. Prediction of maize single-cross performance using RFLPs and information from related hybrids. Crop Sci. 1994;34:20–25.

[6] Haley CS Visscher PM. Strategies to utilize marker quantitative trait loci associations. J Dairy Sci. 1998;81:85–97. 9777515 10.3168/jds.s0022-0302(98)70157-2

[7] Whittaker JC Thompson R Denham MC. Marker-assisted selection using ridge regression. Genet Res. 2000;75:249–252. 10816982 10.1017/s0016672399004462

[8] Meuwissen TH Hayes BJ Goddard ME. Prediction of total genetic value using genome-wide dense marker maps. Genetics. 2001;157:1819–1829. 11290733 10.1093/genetics/157.4.1819 PMC1461589

[9] Gianola D Fernando RL Stella A. Genomic-assisted prediction of genetic value with semiparametric procedures. Genetics. 2006;173:1761–1776. 16648593 10.1534/genetics.105.049510 PMC1526664

[10] de los Campos G Gianola D Rosa GJM Weigel KA Crossa J. Semi-parametric genomic-enabled prediction of genetic values using reproducing kernel Hilbert spaces methods. Genet Res. 2010;92:295–308. 10.1017/S0016672310000285 20943010

[11] Pérez P de los Campos G Crossa J Gianola D. Genomic-enabled prediction based on molecular markers and pedigree using the Bayesian linear regression package in R. Plant Genome. 2010;3:106–116. 21566722 10.3835/plantgenome2010.04.0005 PMC3091623

[12] Zhao Y Gowda M Liu W et al Accuracy of genomic selection in European maize elite breeding populations. Theor Appl Genet. 2012;124:769–776. 22075809 10.1007/s00122-011-1745-y

[13] Howard R Carriquiry AL Beavis WD. Parametric and nonparametric statistical methods for genomic selection of traits with additive and epistatic genetic architectures. G3 (Bethesda). 2014;34:1027–1046. 10.1534/g3.114.010298 PMC4065247 24727289

[14] Crossa J Pérez-Rodriguez P Cuevas J et al Genomic selection in plant breeding: methods, models, and perspectives. Trends Plant Sci. 2017;22:961–975. 28965742 10.1016/j.tplants.2017.08.011

[15] Christensen OF Madsen P Nielsen B Ostersen T Su G. Single-step methods for genomic evaluation in pigs. Animal. 2012;6:1565–1571. doi:10.1017/S1751731112000742. 22717310

[16] Legarra A Aguilar I Misztal I. A relationship matrix including full pedigree and genomic information. J Dairy Sci. 2009;92:4656–4663. doi:10.3168/jds.2009-2061. 19700729

[17] Xavier A Hall B Hearst AA Cherkauer KA Rainey KM. Genetic architecture of phenomic-enabled canopy coverage in Glycine max. Genetics. 2017;206:1081–1089. 28363978 10.1534/genetics.116.198713 PMC5499164

[18] Purcell LC. Soybean canopy coverage and light interception measurements using digital imagery. Crop Sci. 2000;40:834–837.

[19] Xavier A Muir WM Rainey KM. Assessing predictive properties of genome-wide selection in soybeans. G3 (Bethesda). 2016;6:2611–2616. 27317786 10.1534/g3.116.032268 PMC4978914

[20] Habier D Fernando RL Dekkers JCM The impact of genetic relationship information on genome-assisted breeding values. Genetics. 2007;177:2389–2397. doi:10.1534/genetics.107.081190. 18073436 PMC2219482

[21] VanRaden PM Efficient methods to compute genomic predictions. J Dairy Sci. 2008;91:4414–4423. 18946147 10.3168/jds.2007-0980

[22] Kaufman L Rousseeuw PJ. Finding Groups in Data: An Introduction to Cluster Analysis. New York, NY: Wiley; 1990.

[23] MacQueen J Some methods for classification and analysis of multivariate observations. In: Le Cam LM Newman J eds. Proceedings of the Fifth Berkeley Symposium on Mathematical Statistics and Probability Berkeley, CA: University of California Press; 1967:281–297.

[24] Kassambara A. Practical Guide to Cluster Analysis in R: Unsupervised Machine Learning (Multivariate Analysis). Vol 1 Grenoble, France: STHDA; 2017.

